# What are the reasons for clinical network success? A qualitative study

**DOI:** 10.1186/s12913-015-1096-5

**Published:** 2015-11-05

**Authors:** Elizabeth McInnes, Mary Haines, Amanda Dominello, Deanna Kalucy, Asmara Jammali-Blasi, Sandy Middleton, Emily Klineberg

**Affiliations:** Nursing Research Institute – St Vincents Health Australia (Sydney) and Australian Catholic University, DeLacy Building, 379 Victoria Road, Darlinghurst, NSW 2010 Australia; School of Nursing, Midwifery & Paramedicine (NSW & ACT), Australian Catholic University, North Sydney, 2060 NSW Australia; Sax Institute, Level 13, Building 10, 235 Jones Street, Ultimo, NSW 2007 Australia; School of Public Health, The University of Sydney, Sydney, 2006 NSW Australia; NSW Kids and Families, 73 Miller Street, North Sydney, NSW 2060 Australia; Sydney Medical School, The University of Sydney, Sydney, 2006 NSW Australia

**Keywords:** Clinical networks, Outcomes, Stakeholder views, Qualitative

## Abstract

**Background:**

Clinical networks have been established to improve patient outcomes and processes of care by implementing a range of innovations and undertaking projects based on the needs of local health services. Given the significant investment in clinical networks internationally, it is important to assess their effectiveness and sustainability. This qualitative study investigated the views of stakeholders on the factors they thought were influential in terms of overall network success.

**Method:**

Ten participants were interviewed using face-to-face, audio-recorded semi-structured interviews about critical factors for networks’ successes over the study period 2006–2008. Respondents were purposively selected from two stakeholder groups: i) chairs of networks during the study period of 2006–2008 from high- moderate- and low-impact networks (as previously determined by an independent review panel) and ii) experts in the clinical field of the network who had a connection to the network but who were not network members. Participants were blind to the performance of the network they were interviewed about. Transcribed data were coded and analysed to generate themes relating to the study aims.

**Results:**

Themes relating to influential factors critical to network success were: *network model principles; leadership; formal organisational structures and processes; nature of network projects; external relationships; profile and credibility of the network*.

**Conclusions:**

This study provides clinical networks with guidance on essential factors for maximising optimal network outcomes and that may assist networks to move from being a ’low-impact’ to ‘high-impact’ network. Important ingredients for successful clinical networks were visionary and strategic leadership with strong links to external stakeholders; and having formal infrastructure and processes to enable the development and management of work plans aligned with health priorities.

## Background

Clinical networks have been established in several countries to improve patient outcomes and processes of care by implementing a range of innovations and undertaking projects based on local health needs. In Australia, a number of states have now established clinical networks and all aim to engage clinicians in improving patient care and making system-wide changes [1, 2]. The term clinical network describes many different models of networks, from those focused on service delivery systems to informal communities of practice [3–6]. In this study, the term clinical networks refers to voluntary multidisciplinary networks of clinicians that aim to improve clinical care and service delivery using a collaborative approach to identify patient and health service need and to implement strategies to improve quality of care and patient outcomes [1].

In the state of New South Wales, clinical networks are funded by the Agency for Clinical Innovation which sits within the state-based Ministry of Health. These networks, which are multidisciplinary in composition, have a single clinical or disease focus (for example burn injury, nuclear medicine, aged care, stroke) and largely rely on clinicians providing voluntary, often out of hours work. This is similar to network models in Europe, the UK and USA [3–6]. In collaboration with the Agency for Clinical Innovation, clinical networks are primarily focused on projects to improve processes and quality of care across metropolitan and rural NSW. They work in partnership with local health districts, clinicians, consumers, researchers, professional organisations and non-government organisations (www.aci.health.nsw.gov.au/networks). Clinical networks in NSW also provide expert advice to the state-based Ministry of Health in relation to the development, design and implementation of models of care and quality of care improvements. With significant investment in such networks in Australia and internationally, it is important to assess their progress and impact.

While there is some evidence that clinical networks can improve the delivery of healthcare, a recent systematic review of qualitative and quantitative studies has found few studies that have rigorously evaluated the impact and effectiveness of clinical networks [Brown B, Patel C, McInnes E, Mays N, Young J, Haines M]: The effectiveness of clinical networks in improving quality of care and patient outcomes: a systematic review of quantitative and qualitative studies, submitted. The extant published studies are largely descriptive, mainly focused on a single network and have no qualitative or quantitative examination of critical factors associated with network impact or distinguish successful from unsuccessful networks [1, 7]. A previous qualitative study investigated stakeholders’ views on desirable network outcomes and the barriers and facilitators to establishing effective networks [8]. Results informed the design of the measures in a retrospective mixed-methods evaluation of 19 clinical networks [1]. Other qualitative or mixed-method studies of networks have focused on: stakeholder perceptions about the key factors relating to indicators of network effectiveness and sustainability [2]; how managed health care networks in the English National Health Service address ‘wicked problems’ in health care and associated enablers [9]; and the optimum governance structures for networks [10]. A Swedish qualitative study investigated the factors that may differentiate successful from unsuccessful network development, and found the critical factors to be professional dedication; legitimacy and confidence in network leaders, personnel and external collaborators and effective management systems [11].

**Fig. 1:**
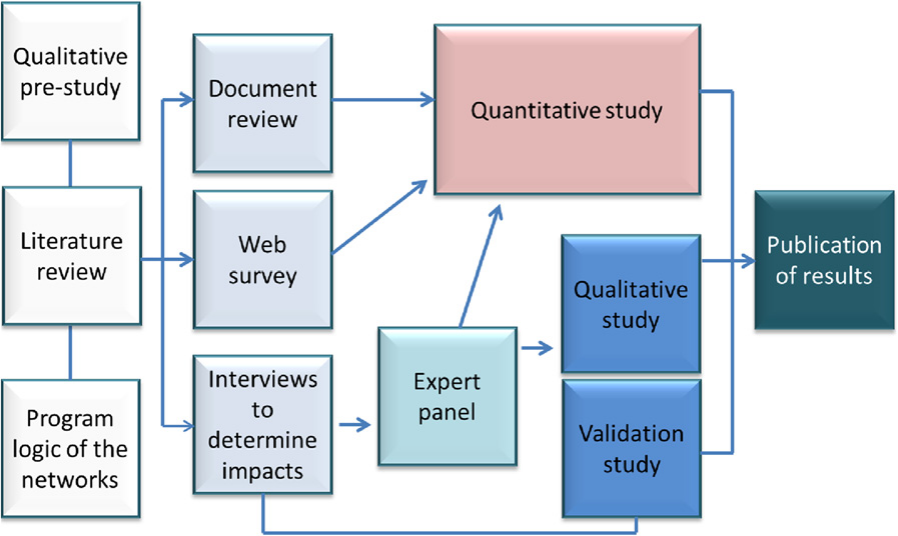
Study overview

The qualitative study reported here was conducted in 2013 towards the end of a mixed-methods retrospective study that evaluated the impact of 19 NSW clinical networks that were in operation during the period 2006–2008 which was an early stage in the establishment of clinical networks in NSW [1] (see Fig. 1). Figure 1 details the sequencing of the study phases including the two qualitative sub-studies. The first one in 2012 [8], was conducted to investigate what stakeholders (different participants to those interviewed for the study reported here) believed were desirable network outcomes and their views on the barriers and facilitators that were likely to influence the establishment of effective networks. The study reported here, using a different sample, was conducted to investigate what participants believed were critical factors in helping to achieving actual network impacts. Impacts that were evaluated had occurred before 2011 and were measured in terms of improvements in quality of care and facilitation of system-wide change. *Improvement in quality of care* was defined as an improvement to the safety, effectiveness, appropriateness, accessibility, efficiency and patient-centred nature of care and *system-wide change* defined as the adoption of network quality improvement initiatives on a larger scale across the wider health system [1]. In the main study, these variables were quantified as median ratings as determined through independent assessment of documentary evidence by members of an international Expert Panel, comprising of five national leaders in health policy and implementing system-wide change, using an expert panel method adapted from the RAND appropriateness method [1]. Briefly, panel members independently rated each network on the impact of its projects on quality of care and system-wide change [1], Dominello A, Yano EM, Klineberg E, Redman S, Craig J, Brown B, Haines M: Measuring the impact of diverse quality improvement activities of clinical networks using the EXpert PANel Decision (EXPAND) method, unpublished.

The aim of the qualitative study reported here was to identify the views of stakeholders internal and external to clinical networks regarding influential factors in NSW clinical networks, in terms of achieving impacts on quality of care and system-wide change during the period 2006–2008. The study was conducted to assist with interpretation of the explanatory quantitative results arising from the main study [1]. In addition, understanding the critical ingredients that have enabled networks to achieve significant impacts is important for providing insights into what is required for overall network success and will provide valuable information for the future planning and optimal development of clinical networks.

## Method

### Research design

Qualitative descriptive study [12] using in-depth semi-structured interviews.

### Sample

#### Network sample

Six networks that had been in operation during the period 2006–2008 were sampled on the basis of their rating by an Expert Panel as high-impact (*n* = 2); moderate-impact (*n* = 2); and low-impact (*n* = 2). Details of the Expert Panel rating process are available in Dominello et al. [Dominello A, Yano EM, Klineberg E, Redman S, Craig J, Brown B, Haines M: Measuring the impact of diverse quality improvement activities of clinical networks using the EXpert PANel Decision (EXPAND) method, unpublished]. When sampling the high and moderate-impact networks, we selected a range of networks that had different types and number(s) of impacts and combinations of impacts (for example, development of models of care; educational programmes; clinical protocols).

#### Participant sample

A purposive maximum variation sampling approach was used to recruit participants from two groups (internal and external stakeholders). Eligible participants were 18 years of age or over and able to give informed consent. Internal stakeholders from each of six networks were either a current or past chair who had held that post during the 2006–2008 study period. The sample interviewed from this group was six, that is one from each network.

External stakeholders were recognised experts in the clinical field of the network or who were connected to the network, through network membership or through their connections with the local health services in which the clinical network operated. These individuals were not involved in leading network initiatives but chosen for their ability to reflect on the achievements of the networks in broader national and clinical contexts. They held senior leadership positions in professional colleges or health policy and planning, or were senior clinicians, for example heads of clinical departments related to the clinical focus of the network. One stakeholder from this group was interviewed for each of the high and moderate-impact networks. External stakeholders associated with the two low-impact networks were not selected for interview because ethics requirements stipulated that these networks must remain confidential. In addition, there were no examples of network impact to focus discussion.

### Procedure

Once selected, all potential participants were sent an advanced letter signed by the investigators and the Chief Executive of the Agency for Clinical Innovation, the body responsible for engaging clinicians and designing and implementing new models of care in NSW. This letter explained the research aims, informed potential participants that they met the eligibility criteria and that a researcher would contact them to ascertain whether they were agreeable to participate. In the case where a selected potential interviewee was not agreeable or available, another eligible individual was selected from that network. A phone call was made one week after the letters were sent to see if those selected were agreeable to participate, confirm that participants had sufficient knowledge of the network to comment on its impacts (external stakeholders only) and make appointments for the interview. While all participants were aware of the name of the network(s) under discussion and what impacts the network had attained, they were blinded to the Expert Panel rating of the network.

### Data collection

After obtaining informed consent, individual, semi-structured, audio-recorded, face-to-face interviews using a topic guide were conducted at a place convenient to the interviewee. The interviews were of approximately 20–30 minutes duration. For all interviewees, the main focus of the interviews was to identify views on the critical factors they believed were needed for clinical networks to enable impact on quality of care and system-wide change.

The interview commenced with an open exploration of reasons for achieving impacts. For participants from or connected to high-moderate impact networks, a diagrammatic representation of each impact relating to improvements in quality of care or system-wide change achieved by the network was used to facilitate discussion on reasons for overall network success. For those participants from low-impact networks the main focus of discussion was barriers to success. All participants were asked for their views on how future networks can maximise success.

### Data analysis

Audio-recorded, anonymised interviews were transcribed verbatim to produce transcripts of narrative text for thematic analysis. The coding frame was developed iteratively as two researchers read the transcripts and developed codes relating to factors that were believed to influence network ability to achieve impacts. Recurrent themes were noted including those covered by the topic guide and others which emerged from participant feedback. Excerpts from the transcripts were allocated to these codes. The coding frame and themes were further refined by examining differences and convergences in the data. Participant quotes were used to illustrate meaning in the themes and accompanying narrative summaries.

Analysis was conducted alongside the interview process, facilitating reflection on the emerging themes to be probed further in later interviews. The final sample size was determined by saturation of themes, that is, when no new insights were identified in the data. Thematic saturation was reached after interviewing 10 participants associated with or connected to the six networks sampled (six network chairs and four external stakeholders).

### Ethics

Ethics approval was granted by the Human Research Ethics Committee of the University of Sydney and ratified by the Australian Catholic University Ethics Committee. All participants were informed of the study objective and were free to participate or withdraw from the study at any point. Participants gave written consent to be interviewed and for the interviews to be audio-recorded. Recordings and transcripts were coded so that the origin of each one could not be identified.

## Results

The findings are presented under the six themes pertaining to network success resulting from our analysis. These were: network model principles; leadership; formal organisational structures and processes; nature of network projects; external relationships; profile and credibility of the network.

### Network model principles

Participants identified the importance of network principles as providing a platform for the development of high-impact projects. The formation of clinician-led networks in NSW in 2002, was predicated on the principles of multidisciplinary membership, collaboration, and engagement in health system innovation [13]. Participants remarked that these principles had underpinned the development of strategic network project objectives focused on addressing local and statewide health system needs that, prior to the establishment of clinical networks, had not been satisfactorily addressed because of lack of multidisciplinary input: ‘*What gets things up is the whole network package’.* What this ‘package’ added to the pre-existing status quo, was a mechanism by which multi-disciplinary groups of clinicians could initiate and drive collaborative projects focused on quality improvement and improving patient outcomes related to their clinical specialty, using a ‘bottom-up’ push.

The principle of multidisciplinary collaboration was also believed to foster goodwill amongst clinicians and had fostered an ethos which was ‘*collaborative rather than antagonistic’*. This was thought to have resulted in two positive effects: i) reinvigoration of clinician interest in participating in projects to improve quality of care and facilitate health system changes, and ii) provision of a forum for developing projects that addressed patient health needs in collaboration with the state-based Ministry of Health and other external partners. This is summarised in a quote from a network chair:

*Networks make it easier for change and broad planning, it is the composition, structure and organisation of networks that has enabled achievements to take place*.

It was noted that prior to the establishment of networks, attempts to effect large-scale clinician-driven changes in processes and quality of care had been unsuccessful. This was thought to be because clinicians interested in health system innovation lacked a formal mechanism for:

*Joint hypothesis development with colleagues and for conversations to occur. There were [health system] gaps that had been looked at but not successfully.*

Overall, participants stated their belief that networks that embraced the principles of multidisciplinary membership and collaboration, and engagement with health system innovation, were those that were more successful in initiating and executing projects with high-level impacts.

### Leadership

Leadership, including both clinical and strategic leadership by the network chair and day-to-day management by the network manager, was seen as a critical success factor by all participants and was also thought to be of more importance than resources: ‘*The right people are more important than financial resources’.*

In relation to the network chairs, core attributes were being visionary, strategic and trusted by internal and external stakeholders. The ability of the network chair to engage the multidisciplinary clinical workforce in network initiatives, collaborate with external stakeholders, and to drive the implementation of network objectives was seen as critical for providing the foundation for the delivery of high-impact projects and creating a momentum towards successful network outcomes. For network chairs (and network managers) skills in negotiation and relationship building were regarded as essential by all participants. These skills were thought to bring the ‘*outliers into the fold’* (‘outliers’ referring to those not involved in networks) and to enable connection with key partners in academic, professional and policy spheres, thus ‘*building a broad base of support from which to facilitate achievements and significant impacts’*.

Both groups of participants expressed the view that the role played by the network chair was critical in the early stage of network establishment:

*New networks need good leadership but eventually the organisation has to have a strength of its own in order to be successful’*.

It was remarked that beyond the establishment phase, with a good network manager in place, the network should become less dependent on the network chair. Some participants from both groups interviewed, believed that those networks that were highly dependent on the activities of the network chair were less likely to have impacts because the network became a ‘*one-person show’*, with ‘*limited reach’* as well as burning-out the network chair.

Participants were unanimous about the critical importance of the role of the network manager in contributing to network success. Participants from the high- and moderate- impact networks frequently attributed the success of their network to the work and skills of the network manager. As well as project management, communication, organisational, coordination and leadership skills network managers also needed high credibility, be able *‘to talk the language of networks’*, that is to communicate the vision and principles of networks to a range of clinical and policy stakeholders, and to have good interpersonal skills. To lay the groundwork for later success the network manager had to ‘*hit the ground running’* in the early stages of a network’s development. One participant attributed the success of their network to the activities of their network manager in the early stages of the network’s history:

*They built links; met with all key players and created formal and informal communication channels between diverse bodies.*

Effective network managers also:

*Provided a hub around the network’s clinical specialty and engaged with key stakeholders and the broader clinical base formally and informally.*

There were two unique aspects of this role that were believed by participants to lay the groundwork for success. One was the ability to ‘*clear a path through the maze of issues’.* This referred to the ability to anticipate and manage operational and project complexities and to negotiate with and involve the right stakeholders at the right time in relation to critical project stages. The other unique aspect was the ability to effectively translate information and ideas to different audiences, namely to clinicians, funders and policy makers. In effect, acting as a knowledge-broker able to ‘*deliver high content knowledge’* to a wide audience and to:

*Sell the project – make internal and external stakeholders feel as though the network initiative is an important, achievable target.*

### Formal organisational structures and processes

Most participants stated that successful networks were strengthened by formal organisational structures and processes and having ‘*solid systems’* in place that facilitated effective planning and communication. These systems included project workgroups focused on project planning and implementation, with broad clinical and consumer representation and meetings with structured agendas and minutes: ‘*Not chat-fests that result in nothing’.* Those networks where the main objective was ‘*just about showing up at meetings and giving a progress report’* were perceived by many as those that made little impact.

Open governance and leadership rotations were regarded as important for securing and maintaining engagement and for ongoing reinvigoration of the network:

*Makes people feel engaged, willing to give solid commitment and feel part of the network’s mission.*

Having a formal link to the state Ministry of Health either directly or through the Agency for Clinical Innovation was considered important for success in a number of ways including opportunities to negotiate resources; ensuring that network initiatives were strategically aligned and for raising the profile of the initiative amongst policy-makers. Establishing effective network communication channels that reached clinical and professional groups enabled the rapid dissemination of network messages and showcasing of network outcomes:

*There is a predictable and solid structure with sophisticated communication systems that facilitate rapid dissemination and updating of information and knowledge.*

This also helped to build awareness of network innovations, attracting interest and aiding spread and up-scale.

### Nature of network projects

This theme referred to characteristics of network projects that were most likely to result in a positive impact. These characteristics included: i) those that addressed systematically identified patient health needs and ii) those that aligned to the state-based Ministry of Health strategic plans. As well as being targeted and achievable, successful projects were those that were:

*Easy to think through with a defined group involved and which have something in it for everyone*.

Acting on the ‘*penny drops’* factor in terms of project timing, seizing opportunities for funding and broad engagement with health services, and gaining the attention of the Ministry of Health through innovative work increased project impact and the likelihood that the network would achieve successful outcomes. Projects that had ‘*capitalised on internal and external expertise’* and that avoided being overly complex in terms of objective and implementation were also thought to be more successful.

Both groups of participants noted that projects that had not addressed a significant health need, were not aligned with Ministry of Health strategic goal, or were not evidence-based were thought to have minimal impact. A number of participants from both groups noted that network projects focused on introducing complex health system changes, particularly if this had involved health care personnel not represented within the network (for example, primary care or paramedicine) were perceived as less likely to be successful and showing little return for effort. One participant (former Chair) noted that networks that focused on long-term chronic conditions with a low prevalence, yet which resulted in multiple physical, social and psychological problems for the client group were often overshadowed by high-profile diseases (for example, stroke and heart disease) which were perceived to attract more Ministry of Health interest and funding.

### External relationships

Links to academic, professional, policy and clinical organisations external to the network were seen as critical for network success. As remarked by an external stakeholder:

*If there is no acceptance [of clinical networks or projects] in those [external] groups then the impact/successes would not happen.*

Participants from both groups remarked that through these links and relationships (usually fostered by the network chair and network manager) opportunities emerged for i) project up-scale, that is expansion of the reach and scope of the project; ii) social marketing of network achievements and iii) engaging a broader base of clinical, academic and policy-maker groups. Without these opportunities, network projects would have limited reach and impact. Acceptance, engagement and interest by external organisations helped to lift the profile and the credibility of the network, as well as provided an additional vehicle to publicise network achievements and successes:

*My clinical speciality has a very strong craft group with national and international respect and it is this link with my network that has been a significant lever for change and widespread adoption of the initiative resulting in a big impact.*

Relationships with the Ministry of Health, including regular meetings with Ministry of Health representatives were regarded as a key factor in project success: ‘*We were able to successfully petition the Ministry of Health through this link’.* Sometimes relationships with Ministry of Health took a number of years to establish, while for other networks a strong relationship was never achieved. Within a constantly changing health landscape it was thought essential for future networks to have this relationship and to also recognise network limitations: ‘*Networks are limited in how much change they can effect. They can help redesign but not lobby’.*

Networks that had not formed strategic external relationships were thought by some participants to have limited reach and to experience difficulties in facilitating projects that could deliver significant impacts:

*They [the network] do have a strong voice with local hospitals, but not really beyond. It is the relationships with external bodies (professional, academic and health departments) that helps to facilitate and implement projects that will make the bigger impact.*

Ability to effectively engage with external bodies relevant to the network such as government; professional organisations and colleges, was therefore seen as important for maximising network potential to achieve significant long-term impacts.

### Profile and credibility of the network

The profile and credibility of the network was seen as highly important for engaging clinicians and policy-makers, attracting funding and enabling the establishment of strategic external relationships. Some participants believed that the most successful networks had built their credibility and enhanced their profile by achieving a number of minor successes in the early stages of the network’s development. These minor successes of ‘quick wins’ were believed to establish the network’s legitimacy and reputation, attracting interest in the network’s initiatives amongst clinicians and at the level of the health department. This helped to increase the critical mass of the network and create opportunities for future success:

*My network has achieved profile and credibility – this leads to a platform for engaging with important groups and policy-makers in our clinical space.*

## Discussion

Critical factors required for networks to achieve impacts that were nominated by key informants comprised both intrinsic (for example, leadership) and external factors (for example, external relationships). Participants believed the most influential factors for overall network success were (in no particular order of priority): being faithful to *network model principles; leadership* – both network chairs and network managers*; formal organisational structures and processes; nature of network projects; external relationships; profile and credibility of the network*. In part, these findings accord with some of those from the main study, to which the study reported here is related. Multivariable analysis in that study demonstrated that strategic and operational management of a network had statistically significant associations with impact on quality of care; and perceived leadership of the network manager and strategic and operational management of a network had statistically significant associations with impact on system-wide change after controlling for potential confounding factors [Haines M, Brown B, D’Este C, Yano E, Craig J, Redman S, et al. Improving the quality of healthcare in a complex system: A cross-sectional study of the features of successful clinical networks, unpublished].

This study builds on the findings of our previous qualitative study which examined stakeholders’ views about conditions needed in general for the establishment of successful clinical networks, that is without the benefit of hindsight or reflecting on impacts that had occurred [8]. In our previous study, the five key important factors identified by key stakeholders were: *Building relationships; effective leadership; strategic evidence-based work plans; adequate resources;* and *ability to implement and evaluate network initiatives* [8]. However, in the current study, in which participants offered their views on what they thought were the actual critical ingredients that had helped to achieve impacts that had occurred, resources or ability to evaluate network initiatives were not perceived as the most critical factors for network success. Interestingly, resources were regarded by the majority of participants in the study reported here as less important than leadership, and in particular, those from moderate-high impact networks thought that networks do not necessarily have to be ‘well-resourced’ to be highly successful.

Network principles of collaboration and multidisciplinary involvement were regarded as the starting point for developing successful and innovative projects along with achieving some ‘quick wins’ in the early days of network establishment. Others have noted that early successes are required to demonstrate network potential and to attract strong clinician engagement [14]. Early successes can help to grow a network’s profile and credibility, attracting further opportunities as the network becomes an established and recognised ‘voice’. The development of a ‘critical mass’ is a major factor in the successful implementation of projects, rather than the efforts of a single individual [15]. Network capacity, through leadership and organisation, to engage ‘the outliers’, that is clinicians who were initially sceptical of clinical networks, was regarded as a pivotal for laying the groundwork for success.

In our study, the stand-out ‘critical ingredient’ for network success was the roles of the network chair and network manager. In a study of managed networks, success could only occur if the network was driven by a “small energised, ‘hybrid’ leadership team, containing a mix of doctors, nurses and managers” [9]. A qualitative study of Swedish networks also found that confidence in network leaders was regarded as a critical factor for network development [11]. In terms of achieving significant impacts these roles are particularly important for innovation spread and up-scale [16]. In our study, charismatic and visionary network chairs who could negotiate with the Ministry of Health, particularly in the early stages of a network’s history, were perceived as kick-starting the network on the road to success. These individuals had attributes indicative of a transformational style of leadership [17]. These attributes included possessing knowledge of complex systems, ability to influence peers and to bring together diverse groups of people across disciplinary and organisational boundaries to form positive and productive alliances. These types of clinical leaders have been termed ‘strategic clinicians’ [15] and represent clinicians who ‘think more managerially and strategically’ [15]. However it was also noted that those networks who mainly depended on a network chair to be the primary driving force, risk having limited impacts.

A highly effective network manager was seen as integral for a high-impact network. One of the most notable features of this role mentioned by some participants was that of knowledge-broker, specifically someone who could effectively translate information and ideas to policy-makers, funders and clinicians and who proactively shared knowledge. Other researchers have remarked that the knowledge-brokering role is a key factor in fostering health service innovation [2]. Middle managers (such as network managers) have the potential to bridge information gaps that might otherwise impede innovation implementation [17, 18]. Individuals in this role can use their ‘in-between’ position to support innovation through ‘getting the right knowledge into the right hands, at the right time’ [17]. They are the constant link between operational and strategic contexts and information flows. Brokers can support the transfer of specialised knowledge between groups, increasing cooperation and by introducing ‘good ideas’ from one isolated setting into another [19]. In this case, clinical network innovations are the ‘good ideas’ with an effective manager able to facilitate that spread of network innovations within and across health services. The essence of effective network leadership appeared to hinge on the ability of the network chairs and network managers to span both discipline and sector boundaries and to champion network agendas and associated projects, linking networks to key stakeholder organisations such as professional colleges and policy agencies to ensure buy-in and the spread of network innovations. The importance of the role of ‘boundary spanners’ [19] in this case referring to the building of relationships and connections between health and policy organisations, has been highlighted in other studies, as has the importance of having organisational champions particularly in the early phases of innovation spread and adoption [16].

Formal organisational structures and processes with communication systems, to support the development of well-designed work plans and implementation of projects, was another nominated reason for network achievements. In another study, effective management systems have been similarly identified as essential for optimal network development [11]. In our study, participants who were connected to more than one network remarked that networks which were formally organised and managed were more able to readily achieve their objectives, than networks with less formal structures. The implementation of formal systems was largely dependent on the network manager and seen as increasing the network’s capacity to respond to changing policy environments. Others have also noted that formal organisational structures and governance enable networks to maximise their achievements [7, 10]. The fit between Ministry of Health agendas and network projects was also an essential ingredient as this facilitated buy-in in the broader health sector and meant that project implementation could capitalise on the current environmental or policy receptivity to the innovation. However, it was expressed by some in the low-impact networks that networks which focused on ‘unpopular’ diseases, defined as where patients live for a long time with a chronic condition would always be judged as less successful because they were granted less resources which in turn meant that these networks could only mount small-scale, less ambitious projects.

External strategic relationships and partnerships – governmental, academic and professional – were seen as instrumental for endorsing and disseminating network innovations, thus providing a stimulus for project uptake outside of the network, increasing reach and maximising impact. When influential others within professional and academic networks have negative opinions of an innovation, it is less likely to be adopted [14]. Having external relationships often relied on the connections of network chairs and network managers and their championing of network projects outside of, as well as within the network. In other studies, it has been noted that networks that are most successful have been observed to effectively reach beyond clinical and organisational boundaries and link across policy, managerial and clinical sectors [7, 9].

In terms of future research, examination of the role, style of working and attributes of network managers would be illuminating. The role of the ‘middle manager’ in effecting change and how top managers leverage off middle managers’ influence in relation to the healthcare innovations of clinical networks are under-researched topics [20–22]. Other research could focus on network governance configurations to investigate whether formally organised networks are more successful than those that are less formally organised and lastly, studies of leadership agency and knowledge transfer to understand the relationship between groups involved in increasing awareness and endorsement of clinical network innovations [20].

### Strengths and limitations

The strengths of our study include the maximum variation sampling strategy that ensured that multiple perspectives were captured through in-depth interviews of key informants related to differently performing networks. The sample included both those directly involved in networks at the time period of interest and those who were recognised experts in the clinical field of the network and were not directly involved in the day to day work of networks, but who were highly knowledgeable about the work of networks. Although some participants had a previous connection with clinical networks and others had knowledge of the network’s activities through their professional or policy positions, these participants were the best placed to provide views on critical ingredients for network impacts. While the sample was small (*n* = 10) information-rich data meant that saturation was achieved. All respondents were blinded to network rating. In keeping with the research method and semi-structured interview technique, interviewees were free to raise any issue that they felt was relevant to the topic under investigation. As a result, it is believed that the information gathered was reflective of genuine concerns and views. A limitation is that the sampled participants were asked about achievements that had occurred during the period 2006–2008. However, participants were selected on the basis of having in-depth knowledge of the network at that time and some were still strongly connected to the network. In addition, it may be that the interviewees expressed publicly acceptable viewpoints and that each network may have their own unique culture that may have influenced responses of participants’ directly involved in networks. However, the interviews were anonymised and confidential in line with ethics requirements and a range of views were expressed from all participants. In terms of transferability of results, clinical networks in NSW have particular features, such as being based on voluntary groups of multidisciplinary clinicians and operate within a specific political and health service environment. The findings reported here may therefore be most relevant for similar networks and may not be generalizable to other clinical networks.

## Conclusion

This qualitative study provides new insights on the critical ingredients needed for clinical networks to achieve successful health system and quality improvement outcomes. The factors considered by external and internal stakeholders of critical importance for ensuring high-impact deliverables were highly effective and well-connected network chairs and network managers, well-designed and strategically aligned projects and the building and fostering of key external relationships with professional and policy organisations. In the absence of these factors, networks may achieve little or no impact.

## References

[1] Haines M, Brown B, Craig J, D’Este C, Elliott E, Klineberg E (2012). Determinants of successful clinical networks: the conceptual framework and study protocol. Implement Sci.

[2] Cunningham F, Ranmuthugala G, Westbrook J, Braithwaite J (2012). Net benefits: assessing the effectiveness of clinical networks in Australia through qualitative methods. Implement Sci.

[3] Hamilton KE, Sullivan FM, Donnan PT, Taylor R, Ikenwilo D, Scott A, et.al. A managed clinical network for cardiac services: set-up, operation and impact on patient care. International J Integrated Care. 2005;5:1–13. 10.5334/ijic.135 PMC1395516 16773161

[4] Laliberte L, Fennell ML, Papandonatos G (2005). The relationship of membership in research networks to compliance with treatment guidelines for early-stage breast cancer. Med Care.

[5] Tolson D, McIntosh J, Loftus L, Cormie P (2007). Developing a managed clinical network in palliative care: a realistic evaluation. Int J Nurs Stud.

[6] Gale C, Santhakumaran S, Nagarajan S, Statnikov Y, Modi N. Impact of managed clinical networks on NHS specialist neonatal services in England: population based study. BMJ. 2012; 344: e2105 10.1136/bmj.e2105 PMC3318112 22490978

[7] Addicott R, McGivern G, Ferlie E (2006). Networks, organizational learning and knowledge management: NHS cancer networks. Public Money Manag.

[8] McInnes E, Middleton S, Gardner G, Haines M, Haertsch M, Paul C (2012). A qualitative study of stakeholder views of the conditions for and outcomes of successful clinical networks. BMC Health Serv Res.

[9] Ferlie E, Fitzgerald L, McGivern G, Dopson S, Bennett C. Making wicked problems governable? The case of managed networks in health care. 1st ed. Oxford: Oxford University Press; 2013.

[10] Willem A, Gemmel P (2013). Do governance choices matter in health care networks? An exploratory configuration study of health care networks. BMC Health Serv Res.

[11] Ahgren B, Axelsson R (2007). Determinants of integrated health care development: chains of care in Sweden. Int J Health Plann Manag.

[12] Sandelowski M (2000). Whatever happened to qualitative description?. Res Nurs Health.

[13] Braithwaite J, Goulston K (2004). Turning the health system 90° down under. Lancet.

[14] Spencer A, Ewing C, Cropper S (2013). Making sense of strategic clinical networks. Arch Dis Child.

[15] Pettigrew A, Ferlie E, McKee L (1992). Shaping strategic change ‐ the case of the NHS in the 1980s. Public Money Manag.

[16] Hendy J, Barlow J (2012). The role of the organizational champion in achieving health system change. Soc Sci Med.

[17] Currie G, Gladman J, Lockett A, Waring J, White L (2011). The knowledge brokering role of middle level managers (MLMs) in service innovation: managing the translation gap in patient safety for elderly care. NIHR service delivery and organisation programme.

[18] Birken SA, Lee SY, Weiner BJ (2012). Uncovering middle managers’ role in healthcare innovation implementation. Implement Sci.

[19] Long J, Cunningham F, Braithwaite J (2013). Bridges, brokers and boundary spanners in collaborative networks: a systematic review. BMC Health Serv Res.

[20] Cunningham FC, Ranmuthugala G, Plumb J, Georgiou A, Marks D, Westbrook J, et al. Social-professional networks of health professionals: A systematic review. Sydney: Centre for Clinical Governance Research, Australian Institute of Health Innovation, University of New South Wales, 2010.

[21] McDonald R (2014). Leadership and leadership development in healthcare settings - a simplistic solution to complex problems?. Int J Health Policy Manag.

[22] Oliver K, De Vocht F, Money A, Everett M (2013). Who runs public health? A mixed methods study combining qualitative and network analysis. J Public Health.

